# Comments from the leaving Editor-in-Chief

**DOI:** 10.1017/qrd.2025.10017

**Published:** 2026-01-05

**Authors:** Bengt Nordén

**Affiliations:** Department of Chemical and Biological Engineering, Chalmers University of Technology, Gothenburg, Sweden

After five years as Editor-in-Chief of QRB Discovery, it is with great pleasure and anticipation I hand over the command to Professor Dame Carol Robinson.

When *QRB Discovery* was launched in 2020 as an independent journal, its focus was described as biological phenomena that can be analysed from a molecular angle. Biophysics applies methods traditionally used in physics and chemistry to study the living world, from molecules and cells to entire populations of animals and plants. This interdisciplinary approach has a huge number of applications and has the potential to address some of the biggest challenges facing our species and our planet. It is vital that discoveries with the potential to benefit society are published quickly and transparently. The field had been missing a dedicated venue for publishing ground-breaking results – ‘discoveries’ – that signal exciting new directions, rather than traditional comprehensive studies. This was the gap *QRB Discovery* aimed to fill. *QRB Discovery* has published original research both as unsolicited contributions from researchers who report their significant original findings as well as themed collections addressing timely areas of biophysics, such as ‘*Single Molecule Challenges in the 21st Century*’ and ‘*Frontiers of Computational Biophysics*’.

Authors are encouraged to elaborate on the potential consequences and wider impact of their discoveries. If the research is of high quality and it is a sound result that points in an exciting direction – even if the final steps or impact may still be missing – we have published it. This transparency is further extended by publishing open peer review reports. This will, expectantly, promote a more constructive type of review for authors, but it will also contribute to the recognition of reviewers.

It is my hope that the new Editor-in-Chief will continue to develop *QRB Discovery* as a platform for reporting novel, disruptive findings and encourage young researchers to contribute their work. We warmly welcome Carol Robinson to the team of biophysics journals of Cambridge University Press and Assessment.

